# Intraoperative distensibility measurement in POEM for achalasia: impact on patient outcome and associations with other outcome variables at 1-year follow-up

**DOI:** 10.1007/s00464-023-10494-z

**Published:** 2023-10-25

**Authors:** Helge Evensen, Olav Sandstad, Lene Larssen, Milada Hagen, Vendel A. Kristensen, Torgeir Thorson Søvik, Anselm Schulz, Truls Hauge, Asle W. Medhus

**Affiliations:** 1 Department of Gastroenterology, Oslo University Hospital, Oslo, Norway; 2 Faculty of Medicine, University of Oslo, Oslo, Norway; 3 Faculty of Health Sciences, Oslo Metropolitan University, Oslo, Norway; 4 Department of Pediatric and Gastrointestinal Surgery, Oslo University Hospital, Oslo, Norway; 5 Department of Radiology and Nuclear Medicine, Oslo University Hospital, Oslo, Norway; 6 Department of Diagnostic Physics, Norwegian Imaging Technology Research and Innovation Center (ImTECH), Oslo University Hospital, Oslo, Norway

**Keywords:** Achalasia, Per-oral endoscopic myotomy, LES distensibility

## Abstract

**Background and aims:**

The functional luminal imaging probe (FLIP) can provide measurements of lower esophageal sphincter (LES) distensibility. Studies report that use of intraoperative FLIP examination during peroral endoscopic myotomy (POEM) for achalasia is associated with treatment success, but evidence is limited and inconsistent. The main aim of the present study was to assess associations between intraoperative FLIP values and 1-year outcomes. Additionally, associations between 1-year FLIP measurements and other 1-year outcome variables were studied.

**Methods:**

We performed a single-center prospective study of consecutive achalasia patients treated with POEM with a standardized 1-year follow-up. The inclusion period was from June 2017 to January 2020. We compared 1-year outcomes (FLIP measurement values, Eckardt score (ES), reflux esophagitis, timed barium esophagogram (TBE), and lower esophageal sphincter resting pressure (LES-rp)) in patients with and without intraoperative FLIP examination. We also assessed associations between intraoperative FLIP values, 1-year FLIP values, and other 1-year outcomes. Results are given as median (IQR), and non-parametrical statistical analyses were applied.

**Results:**

Sixty-two patients (27 females) with median age 45 years (35–54) were included. Baseline characteristics were similar in patients with (*n* = 32) and without (*n* = 30) intraoperative FLIP examination. In patients with intraoperative FLIP, ES was 2 (1–3) and LES distensibility index (DI) 3.7 (2.6–5.4) after 1 year, compared with ES 2 (1–3) and DI 4.0 (3.1–6.8)) in patients without intraoperative FLIP (ns). Intraoperative DI was not correlated with 1-year ES or DI. One-year DI correlated significantly with 1-year ES (*r*_s_ − 0.42), TBE (*r*_s_ − 0.34), and LES-rp (*r*_s_ − 0.29).

**Conclusions:**

Use of intraoperative FLIP measurements in POEM for achalasia is not associated with improved 1-year outcome, and the clinical value of intraoperative FLIP in POEM for achalasia is questioned. Follow-up FLIP measurements are moderately associated with symptomatic outcome, and may serve as an additional diagnostic modality in post-treatment evaluation.

**Graphical abstract:**

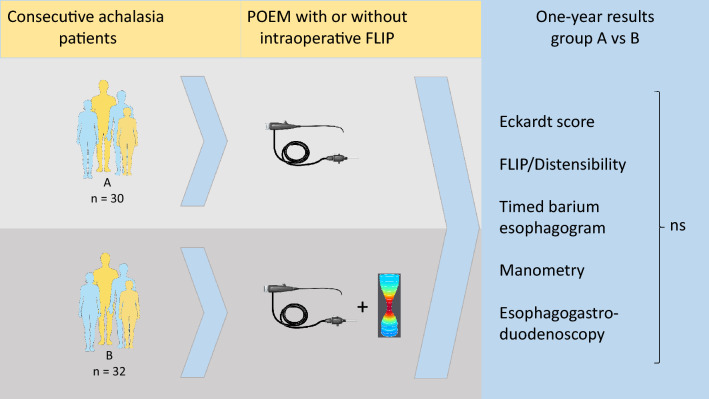

**Supplementary Information:**

The online version contains supplementary material available at 10.1007/s00464-023-10494-z.

The functional lumen imaging probe (FLIP) system consists of a dedicated device with a screen for graphical output, to which a catheter with a distendable balloon is connected. The catheter is typically placed transorally before measurements are performed at standardized balloon fill volumes. The FLIP system offers real-time assessment of cross-sectional area (CSA) in relation to pressure by volume-controlled distention, also known as luminal distensibility. The system has been applied in the upper gastrointestinal tract in different conditions, including distensibility evaluation of the esophageal wall in eosinophilic esophagitis and characteristics of the lower esophageal sphincter (LES) in gastroesophageal reflux disease and achalasia [1]. In achalasia, LES physiology is of particular interest. LES distensibility index (DI), defined as the minimum CSA at LES level divided by distensive pressure, appears to be an important FLIP variable. There is growing optimism concerning the usefulness of the FLIP system in diagnosis, therapy, and follow-up of achalasia [2–6].

The clinical potential of intraoperative FLIP measurements during peroral endoscopic myotomy (POEM) has been addressed in numerous studies, suggesting that the application of intraoperative FLIP measurements may result in a tailored myotomy [3–6]. These studies have reported that DI and CSA measured intraoperatively after myotomy in POEM are associated with post-treatment outcome. In these studies, the association is mainly related to patients’ symptomatic outcome, and objective tests such as timed barium esophagogram (TBE) and high-resolution manometry (HRM) that could potentially complement the evaluation, are in general not included. Also, the protocol for the FLIP procedure is not standardized. Accordingly, interpretation and comparability of the results are difficult and our knowledge on the clinical value of the use of intraoperative FLIP in POEM is thus still limited [7]. FLIP has also been applied in post-treatment evaluation, and it has been suggested that DI might be an important objective outcome in follow-up of achalasia [2, 8, 9]. However, based on the available data on FLIP measurements in achalasia, a more comprehensive evaluation of its clinical role both intraoperatively and in follow-up evaluation is needed.

Our main aim was to compare Eckardt Score (ES) and DI at 40 ml balloon fill volume (DI 40) 1 year after POEM in patients with and without an intraoperative FLIP-tailored myotomy. One-year CSA, TBE, HRM, and esophagogastroduodenoscopy (EGD) were also evaluated. In addition, we aimed to examine associations between intraoperative FLIP and 1-year outcomes and between 1-year FLIP and 1-year outcomes.

## Materials and methods

### Study design and patients

The present study was conducted as a single-center prospective study. All consecutive achalasia patients treated with POEM at Oslo University Hospital during the inclusion period with 1 year follow-up were included. The inclusion period was from June 2017 to January 2020. At the start of the inclusion period, POEM was an established procedure at the institution [10]. Equipment for distensibility measurement by FLIP became available at our hospital in October 2018. Patients treated prior to this were thus evaluated with FLIP only at the 1-year control, whereas patients treated thereafter had both intraoperative and 1-year FLIP performed.

### Data collection

Standardized data from diagnosis, intraoperatively, and at 1-year follow-up were recorded prospectively. Baseline data included demographics, symptom duration, achalasia treatment status, stage, and subtype of achalasia. Standardized 1-year control included ES, FLIP examination, TBE, HRM, and EGD.

#### FLIP system and distension protocol

FLIP measurements were performed intraoperatively and at 1-year follow-up, using a commercially available FLIP system (Endoflip 2.0; Medtronic, Minneapolis, Minn, USA) and 8 cm probes (EF-325N). The catheter was calibrated before measurements. LES confirmation was achieved with 20 ml fill volume. Balloon was filled in a graded approach and monitored for at least 30 s at each fill volume. The FLIP measurements were calculated as the mean value of the final 10 s of registration at each fill volume. Diameter, CSA, intra-balloon pressure, and DI were registered for 30, 40, and 50 ml fill volume at each FLIP procedure. Intraoperatively, premyotomy DI was measured and compared with DI after myotomy. The recorded postmyotomy DI was the final intraoperative FLIP measurement at 40 ml, either after initial myotomy or additional myotomy. If DI value was adequate after initial myotomy, postmyotomy DI was registered as adequate. If the DI value after initial myotomy was inadequate, an additional myotomy was performed, followed by a final FLIP measurement to evaluate postmyotomy DI. Depending on the DI value after additional myotomy (adequate/inadequate), postmyotomy DI was either classified as adequate or inadequate.

#### POEM procedure

Anterior myotomy was the default orientation, posterior myotomy was performed in case of prior POEM. Selective proximal myotomy of the circular layer until 3 cm orally to LES was routinely performed, and distally, a radical myotomy was performed at least 2 cm distal to LES. In case of inadequate postmyotomy FLIP value after initial myotomy, myotomy radicality was visually assessed and additional myotomy was performed by cutting of remaining muscle fibers. POEM procedure time, myotomy length, procedure-related complications, and hospital stay were registered.

#### TBE, HRM, EGD, and ES registration

TBE was performed as described by Neyaz et al. [11]. The ManoScan™ ESO High-Resolution Manometry System (Medtronic, Minneapolis, USA) was applied. HRM was performed and analyzed according to the Chicago classification, v 3.0 [12], and achalasia was classified in subtypes I, II, and III [13]. EGD was performed to evaluate esophagitis according to the Los Angeles classification [14]. Achalasia-related symptoms were registered using ES, ranging from 0 (minimum) to 12 (maximum) [15].

### Definitions and variables

Main outcomes were 1-year DI 40 (mm^2^/mmHg) and ES. Variables achieved with a FLIP fill volume of 40 ml were used in the analyses as these values are assumed to be most reliable [7].

*DI*: LES distensibility index.

*DI 40*: DI at 40 ml fill volume.

*CSA*: Cross-sectional area at LES level.

*Premyotomy DI*: Intraoperative DI 40 after induction of anesthesia, before mucosectomy and myotomy.

*Postmyotomy DI*: Final intraoperative FLIP DI 40 measurement.

*Follow-up DI*: FLIP DI 40 measurement at standard 1-year follow-up.

*Adequate postmyotomy DI*: DI 40 ≥ 4 mm^2^/mmHg *or* increase in DI 40 ≥ 2 compared with premyotomy DI 40.

*Eckardt score:* 0–12 points [15].

*Timed barium esophagogram*: Barium height (cm) at 1 and 5 min [11].

*High-resolution manometry*: LES relaxation pressure (LES-rp, mmHg).

Esophagogastroduodenoscopy: Positive if esophagitis ≥ grade A [14].

*Achalasia stage*: Sigmoid vs non-sigmoid [16].

*Achalasia subtype*: I, II, III [13].

*Symptom duration*: Years from onset of achalasia symptoms.

*Previous treatment*: Prior endoscopic or surgical achalasia therapy.

### Ethics

Data from standard clinical follow-up of patients with achalasia were prospectively included in the study database, which was approved for use in research by the institutional review board at Oslo University Hospital (case number 2016/5437). All patients signed informed consent regarding their willingness to include their data in the study database. The study adheres to the Declaration of Helsinki.

### Statistical analyses

Continuous variables were described with median and interquartile range (IQR). Categorical data were presented as counts and percentages. Crude comparison between pairs of variables were performed using chi-square test (categorical data) or Mann–Whitney Wilcoxon and Wilcoxon signed-rank test (continuous data). Kruskall–Wallis test was applied for comparison between three groups. For correlation analyses, Spearman’s rank-order correlation analysis was used.

All *p* values < 0.05 were considered statistically significant. All analyses were considered exploratory so no correction for multiple testing was done. All analyses were performed using SPSS ver 26 (SPSS, Chicago, IL, USA).

## Results

In total, 62 patients (27 females) with a median age of 45 years (35–54) underwent POEM and were included for further analyses. Of these, 32 patients had an intraoperative FLIP *and* a 1-year FLIP examination, whereas in 30 patients, only 1-year follow-up with FLIP examination was performed. Previous treatment consisted of POEM (*n* = 2), pneumatic dilation (*n* = 7), and botulinum toxin injection (*n* = 1). Three patients with POEM during the study period could not be included due to missing 1-year data. Baseline characteristics, procedural data, complications, and follow-up period were similar in patients with and without intraoperative FLIP examination (Table 1). All complications were Clavien–Dindo grade 1 [17]. There were no complications related to the FLIP examinations.

**Table 1:** Baseline characteristics, procedural data, and follow-up of patients with and without intraoperative FLIP measurements during peroral endoscopic myotomy

	Intraoperative FLIP (*n* = 32)	No intraoperative FLIP (*n* = 30)	*p* value
Gender (F/M)	14/18	13/17	1
Age (years)^a^	47 (37–55)	40 (35–52)	0.31
Achalasia subtype (I/II/III)	6/20/6	5/23/2	0.33
Achalasia stage (sigmoid/not sigmoid)	4/28	8/22	0.21
Previous treatment (yes/no)	5/27	5/25	1
Symptom duration (years)^a^	3.0 (2.0–13.3)	4.5 (2.9–10.5)	0.80
Myotomy (cm)^a^	12 (11–13)	12 (11–14)	0.31
Procedure time (min)^a^	130 (114–141)	125 (115–140)	0.74
Procedural complications (yes/no)	6/26	3/27	0.29
Follow-up (months)^a^	12 (12–13)	13 (12–15)	0.26

*FLIP* functional lumen imaging probe

^a^Median (IQR)

**Fig. 1:**
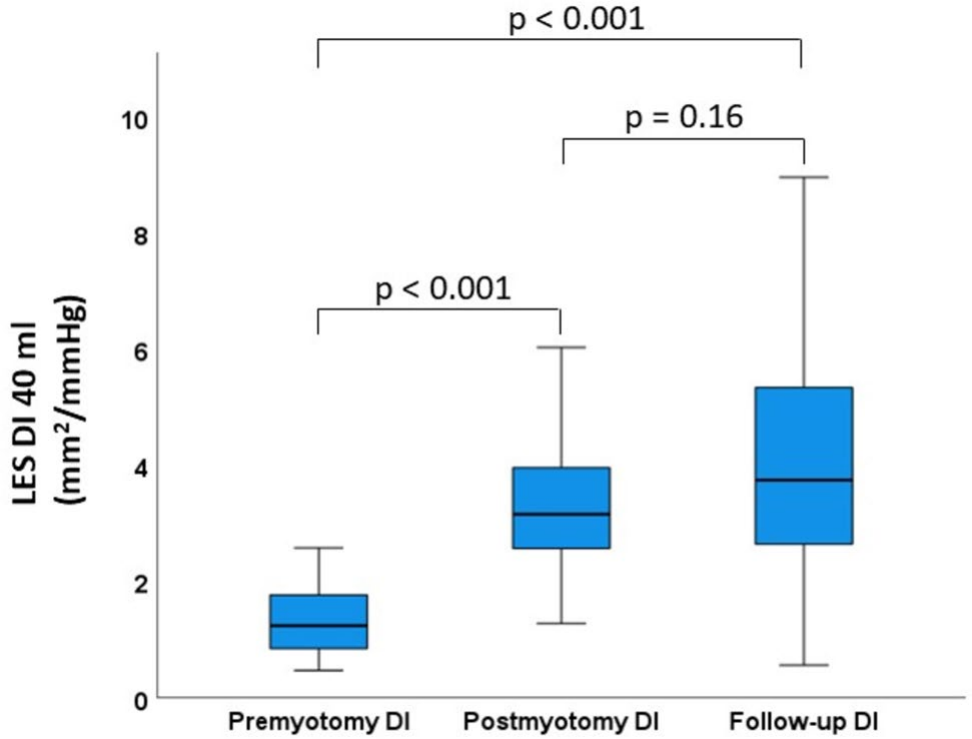
Lower esophageal sphincter distensibility index by functional lumen imaging probe (FLIP) measurement at 40 ml balloon fill volume before myotomy, after myotomy, and at 1-year follow-up in patients with intraoperative FLIP measurements

In patients with intraoperative FLIP measurements, DI 40 increased significantly from a premyotomy value of 1.24 mm^2^/mmHg (0.84–1.77) to a postmyotomy value of 3.16 mm^2^/mmHg (2.54–4.03, *p* < 0.001). At 1-year follow-up, LES distensibility was still significantly higher than before POEM with a DI 40 of 3.75 mm^2^/mmHg (2.61–5.35, *p* < 0.001). Postmyotomy DI 40 and 1-year follow-up DI 40 were similar (Fig. 1).

**Fig. 2:**
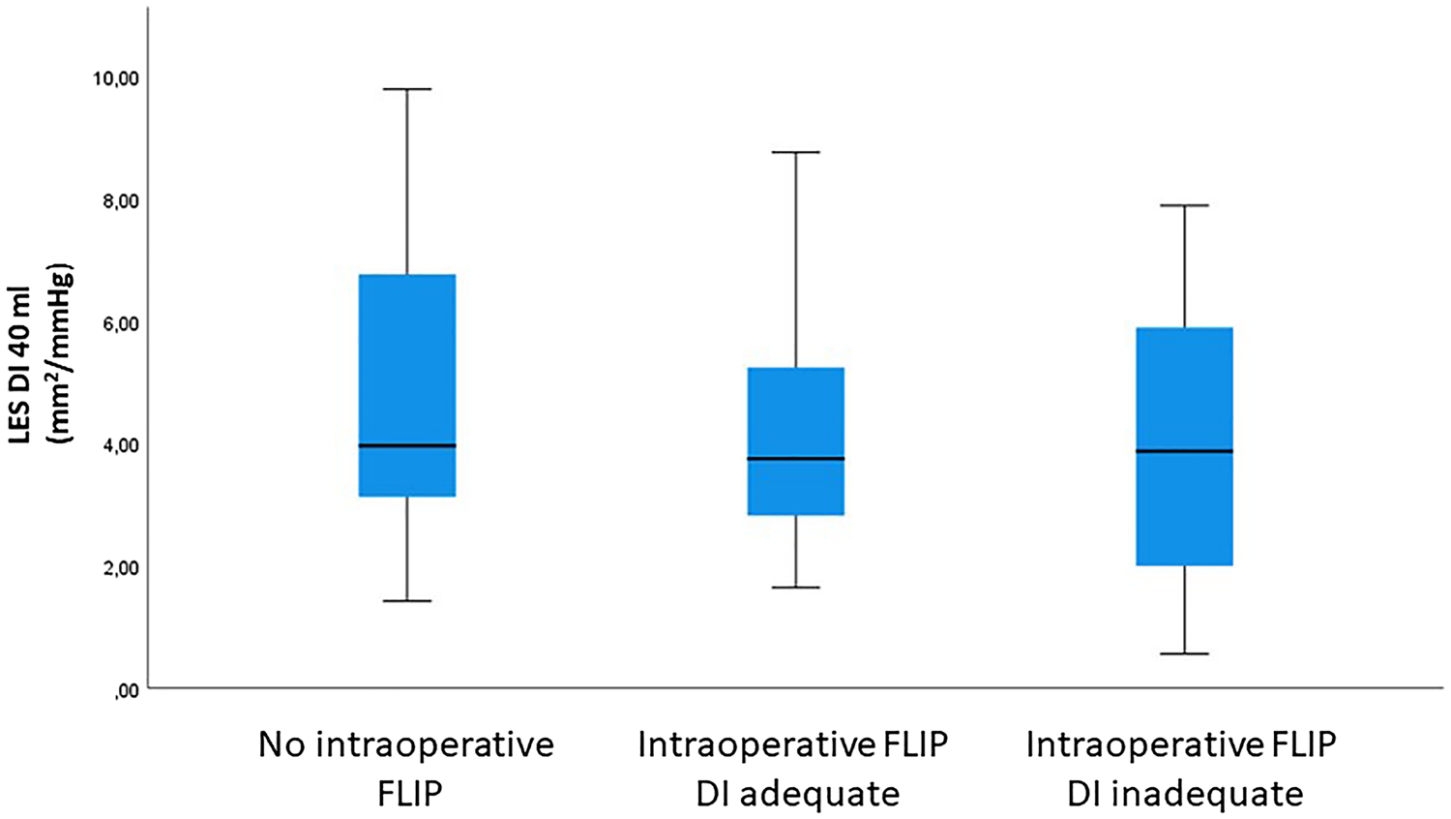
One-year lower esophageal sphincter distensibility index (DI) by functional lumen imaging probe (FLIP) measurement at 40 ml balloon volume in patients with (adequate/inadequate DI) and without intraoperative FLIP measurements

At the 1-year control, ES and DI 40 were comparable in patients with and without intraoperative FLIP examination. One-year CSA, TBE, LES-rp, and frequency of esophagitis were also comparable between the two groups (Table 2). In additional analyses including postmyotomy FLIP value adequacy, 1-year outcomes were similar in patients with adequate intraoperative FLIP value, inadequate intraoperative FLIP value, and in patients without intraoperative FLIP examination (Table 2, Fig. 2). In sensitivity analyses with alternative definitions of postmyotomy adequacy, 1-year outcomes were also similar between patients with and without intraoperative FLIP examination independent of postmyotomy DI values (Supplementary).

**Table 2:** One-year outcomes in patients with and without intraoperative functional luminal imaging probe (FLIP) measurements

(a)	Without intraop FLIP (*n* = 30)	With intraop FLIP (*n* = 32)	*p* value
ES	2 (1–3)	2 (1–3)	0.67
DI 40 (mm^2^/mmHg)	4.0 (3.1–6.8)	3.7 (2.6–5.4)	0.40
CSA 40 (mm^2^)	102.7 (78.1–156.1)	88.1 (72.2–123.2)	0.14
TBE 5 min (cm)	0.5 (0–4.7)	0.0 (0–5.3)	0.92
LES-rp (mmHg)	10.0 (6.7–15.6)^1^	10.0 (6.0–13.0)^1^	0.53
EGD (neg/pos)	20/10	19/13	0.61

(b)	Without intraop FLIP (*n* = 30)	With intraop FLIP, adequate (*n* = 20)	With intraop FLIP, inadequate (*n* = 12)	p value
ES	2 (1–3)	1 (1–3)	2 (2–4)	0.40
DI 40 (mm^2^/mmHg)	4.0 (3.1–6.8)	3.7 (2.8–5.3)	3.9 (1.7–6.2)	0.65
CSA 40 (mm^2^)	102.7 (78.1–156.1)	88.1 (73.2–119.7)	89.9 (42.8–133.8)	0.33
TBE 5 min (cm)	0.5 (0–4.7)	0.9 (0–5.1)	0.0 (0–6.5)	1
LES-rp (mmHg)	10.0 (6.7–15.6)^1^	10 (7.0–12.8)^2^	9.0 (4.4–21.8)^3^	0.82
EGD (neg/pos)	20/10	10/10	9/3	0.31

(a) and (b) median (IQR)

*ES* Eckardt score, *DI 40* distensibiliy at 40 ml fill volume, *CSA 40* cross-sectional area at 40 ml fill volume, *TBE* timed barium esophagogram, *LES-rp* lower esophageal sphincter relaxation pressure, *EGD* esophagogastroduodenoscopy

^1^27; ^2^17; ^3^10

Postmyotomy FLIP values were not correlated with 1-year FLIP values or 1-year ES, TBE, and LES-rp (Table 3).

**Table 3:** Correlations between intraoperative FLIP measurements and 1-year outcomes (*n* = 32)

	1 year outcome				
	DI 40	CSA 40	ES	TBE	LES-rp
Postmyotomy DI 40					
Correlation coefficient	0.081	0.0215	0.0218	0.052	0.011*
*p* value	0.66	0.93	0.92	0.78	0.96
Postmyotomy CSA 40					
Correlation coefficient	0.053**	0.0655**	0.050**	− 0.030**	− 0.003***
*p* value	0.78	0.77	0.79	0.87	0.99

*FLIP* functional lumen imaging probe, *ES* Eckardt score, *TBE* 5 min timed barium esophagogram, *LES-rp* lower esophageal sphincter (LES) relaxation pressure, *DI 40* LES-distensibiliy at 40 ml fill volume, *CSA 40* LES-cross-sectional area at 40 ml fill volume

*n = 27, **n = 31, ***n = 26

Follow-up FLIP measurements were moderately correlated with follow-up ES and less correlated with TBE and LES-rp 1 year after POEM (Table 4). At follow-up EGD, 23 of 62 patients had esophagitis. One-year DI 40 and CSA 40 were similar in patients with esophagitis (3.9 mm^2^/mmHg (3.2–5.4) and 97.1 mm^2^ (83.2–123.4)) compared with patients with no esophagitis (4.0 mm^2^/mmHg (2.4–6.4) and 91.0 mm^2^ (69.0–135.5) (n.s.).

**Table 4:** Correlations between FLIP measurements and ES, TBE, and HRM 1 year after POEM (*n* = 62)

	ES	TBE	LES-rp
DI 40			
Correlation coefficient	− 0.419	− 0.339	− 0.293*
*p* value	< 0.01	0.01	0.03
CSA 40			
Correlation coefficient	− 0.449	− 0.373	− 0.263*
*p* value	< 0.01	< 0.01	0.06

*FLIP* functional lumen imaging probe, *ES* Eckardt score, *TBE* 5 min timed barium esophagogram, *HRM* High-resolution manometry, *LES-rp* Lower esophageal sphincter (LES) relaxation pressure, *DI 40* LES-distensibiliy at 40 ml fill volume, *CSA 40* LES-cross-sectional area at 40 ml fill volume

*n = 54

## Discussion

In the present study, we found that 1-year ES and DI after POEM were similar in patients with and without intraoperative FLIP. Additional 1-year outcomes including TBE, HRM, and EGD were also comparable between the two groups. These findings question the clinical value of performing the FLIP procedure during POEM. On the other hand, 1-year FLIP values were moderately associated with the other 1-year outcome variables, suggesting that FLIP measurements may be an additional useful tool in the post-POEM assessment of patients with achalasia.

There has been considerable interest in the use of intraoperative FLIP during POEM. Ideally, the FLIP system should guide the operator in performing a tailored myotomy of adequate length and completeness to ensure an optimal outcome including improved esophageal clearance, while, importantly in POEM, minimizing the risk of post-therapy gastroesophageal reflux disease. Previous achalasia studies have reported that intraoperative FLIP measurements during POEM may be positively associated with clinical outcome and thus advocated its use [3–6]. The reported effect of intraoperative FLIP on outcome is based on post-treatment symptom registration [3–6], but results are inconsistent, with a recent study demonstrating no correlation between postmyotomy DI and follow-up ES [18]. Furthermore, associations between intraoperative FLIP and treatment success have not been supported by demonstrating associations with objective outcomes such as TBE and LES-rp [4, 18]. There are also methodological limitations to existing studies. Although FLIP measurements performed with 40 ml fill volume are considered most reliable [7], FLIP distention protocols vary across studies. In relevant studies, Ngamruenphong et al. [5] registered FLIP measurements at 30 ml fill volume, Amundson et al. [19] changed the FLIP protocol during their study from 30 to 40 ml fill volume, and Holmstrom et al. [4] used 40 ml fill volume. Also, the reported FLIP variable differed from single-plane CSA [5] and DI [4] measurements to volume-based compliance evaluation [19]. These studies were all retrospective and did not include a follow-up FLIP examination. Moreover, two of the studies [5, 19] did not include a control group without intraoperative FLIP examination.

In contrast, adhering to the recommended FLIP protocol [7], the present study provides prospective data and a standardized 1-year evaluation with repeated FLIP examination, in consecutive patients with and without intraoperative FLIP examination. Interestingly, this evaluation did not indicate that there is a clinical benefit of intraoperative FLIP measurements. There were no differences in 1-year ES and DI between patients with and without intraoperative FLIP, and this was independent of postmyotomy DI adequacy. Additionally, postmyotomy FLIP values were not correlated with follow-up variables. Thus, based on previous and present results, the clinical value of applying FLIP during POEM to improve the myotomy and subsequent outcome is questioned.

In the absence of generally accepted cut-off FLIP values, our definition of postmyotomy DI adequacy at 40 ml fill volume supplements alternative definitions from previous studies. When performing sensitivity analyses on the present data applying the cut-off values of Teitelbaum et al. [20] and Holmstrom et al. [4], respectively, our results remain similar, confirming the lack of associations between intraoperative FLIP and post-POEM outcomes. Thus, the present findings can hardly be explained by the applied definition of postmyotomy DI adequacy.

The lack of associations between intraoperative FLIP and 1-year outcomes may be due to multiple factors. Post-treatment LES remodeling has been proposed as one cause for the discrepancy between registered FLIP values intraoperatively and at follow-up [21]. Intraoperative factors such as capnoperitoneum may be even more relevant, potentially affecting the clinical value of FLIP examination during POEM. Although not always clinically obvious, capnoperitoneum is regularly observed in POEM. Similar to other centers, POEM is performed at our hospital without routine deflation of capnoperitoneum. The resulting increase in intraabdominal pressure may influence intraoperative FLIP measurements and thereby counteract the effect of the myotomy on DI. If so, more valid measurements may be seen after balloon dilation therapy, where capnoperitoneum is not an issue, or during myotomy with routine abdominal deflation by a surgical port or a Veress needle. Alternatively, monitoring bladder pressure during POEM may assist FLIP value interpretation, but will require routine urinary catheterization. Another possible reason that intraoperative FLIP did not result in improved outcomes including follow-up FLIP measurements may be that visual evaluation of myotomy adequacy in itself was sufficient. This latter assumption further questions the need for intraoperative use of FLIP during POEM.

As opposed to postmyotomy DI, follow-up DI was associated with both symptomatic and objective outcomes at 1-year post-POEM follow-up, although the associations were moderate. Post-treatment FLIP may nevertheless be a valuable clinical tool when evaluating treatment outcome and need for retreatment in the individual patient. This is in line with other studies such as the early study by Pandolfino et al. [2]. While DI is a *direct* measure of sphincter opening in relation to pressure, the LES attribute is only indirectly examined with TBE and manometry in achalasia. DI is associated with symptomatic effect, esophageal emptying and LES-rp, and may outperform TBE and manometry in post-treatment achalasia evaluation [8, 9]. However, it can be argued that ES as a cheap, non-invasive and widely used tool that also correlates with FLIP measurements, should be used instead of a FLIP examination in most cases. Furthermore, ES is mainly a symptom-derived score, and for patients, the symptomatic outcome is obviously the most important. At our center, the FLIP evaluation is currently used in the follow-up of challenging clinical cases with inconsistent response across symptomatic and objective follow-up measures. In these cases, FLIP may play a valuable role in post-treatment LES evaluation. Cost–utility data are, however, also needed before a complete evaluation of future use of FLIP examination in achalasia can be performed.

### Strengths and limitations

To our knowledge, this study represents the most systematic evaluation of intraoperative FLIP in POEM for achalasia, owing to the prospective data and the high patient adherence to a standardized and comprehensive 1-year control. Previously, the use of intraoperative FLIP has been evaluated mainly according to patients’ symptoms, while the present study additionally incorporates standard objective outcomes including repeated FLIP measurement after 1 year. However, post-POEM reflux evaluation was based solely on EGD. Furthermore, use of a standardized health-related quality of life (HRQOL) questionnaire would have generated more data on patient-reported outcomes. ES is widely used in achalasia studies, and was applied in the present study due to its simplicity and the close correlations between ES and central HRQOL domains, which has been demonstrated in previous studies [22, 23]. POEM was a well-established procedure before start of patient inclusion, and the two POEM cohorts with and without intraoperative FLIP constituted of consecutive patients.

The single-center design and the performance of POEM exclusively by two experienced endoscopists (LL and HE) ensure a standardized treatment and follow-up of all patients. Although baseline characteristics were similar in the two POEM cohorts, a randomized clinical trial would have been a more optimal design in order to assess the influence of intraoperative FLIP in POEM. Furthermore, the number of patients in our study is comparable to other studies in this particular field, but it is still relatively low. This increases the risk of type 2 errors and limits subgroup analyses on, e.g., previous achalasia treatment, achalasia subtypes and stages.

## Conclusion

The present study demonstrates that intraoperative FLIP measurements are not associated with 1-year subjective or objective outcomes after POEM for achalasia, questioning the clinical value of this procedure. On the other hand, 1-year variables of outcome are moderately associated with 1-year FLIP measurements. This suggests that FLIP measurements might be an additional diagnostic tool that can be applied during follow-up when evaluating treatment efficacy and need for reintervention.

### Supplementary Information

Below is the link to the electronic supplementary material.

Supplementary file1 (DOCX 19 kb)
