# Supplementary material for: Intraoperative distensibility measurement in POEM for achalasia: impact on patient outcome and associations with other outcome variables at 1-year follow-up
Source: Surg Endosc. 2023 Oct 25;37(12):9283–90. doi: 10.1007/s00464-023-10494-z (PMC10709476; doi:10.1007/s00464-023-10494-z)
Supplement: Supplementary file 1 — Supplementary file1 (DOCX 19 kb) [file 464_2023_10494_MOESM1_ESM.docx]

**Supplementary.** One year outcomes in patients with and without intraoperative functional luminal imaging probe (FLIP) measurements.

**a)** Postmyotomy FLIP adequacy: DI40 ≥2.9 mm^2^/mmHg OR ≥2 x premyotomy DI 40(definition by Holmstrom et al (4))

|  | without intraop FLIP  (n = 30) | with intraop FLIP,  adequate (n = 26) | with Intraop FLIP,  inadequate ( n = 6) | p-value |
| --- | --- | --- | --- | --- |
| **ES** | 2 (1-3) | 2 (1-3) | 3 ( 0-4) | 0.80 |
| DI 30 (mm^2^/mmHg) | 3.1 (2.2-4.5) | 2.6 (1.9-4.1) | 1.6 (1.1-2.2) | 0.20 |
| **DI 40** (mm^2^/mmHg) | 4.0 (3.1-6.8) | 3.8 (2.8-5.5) | 2.0 (1.2-5.8) | 0.23 |
| DI 50 (mm^2^/mmHg) | 3.8 (3.3-5.9) | 3.4 (2.9-5.6) | 2.7 (1.0-6.1) | 0.38 |
| CSA 30 (mm^2^) | 51.4 (36.4-71.0) | 46.7 (34.4-66.4) | 24.5 (19.1-39.5) | 0.08 |
| CSA 40 (mm^2^) | 102.7 (78.1-156.1) | 90.9 (74.8-124.2) | 56.2 (26.1-114.7) | 0.15 |
| CSA 50 (mm^2^) | 176.8 (134.8-236.3) | 157.2 (123.2-189.3) | 94.3 (38.3-208.0) | 0.20 |
| TBE 1 min (cm) | 3.0 (0-6.2) | 3.3 (0-5.8) | 0 (0-12.8) | 0.85 |
| TBE 5 min (cm) | 0.5 (0-4.7) | 0.9 (0-5.2) | 0 ( 0-10.1) | 1.00 |
| LES-rp (mmHg) | 10.0 (6.7-15.6)^1^ | 9.0 (5.8-12.7)^2^ | 12.3 (6.0-43.2)^3^ | 0.38 |
| EGD groups neg/pos | 20/10 | 14/12 | 5/1 | 0.34 |

Median (IQR). ^1^ = 27, ^2^ = 22, ^3^ = 5.

**b)** Postmyotomy FLIP adequacy: DI40 4.5 – 8.5 mm^2^/mmHg(definition by Teitelbaum et al (20))

|  | without intraop FLIP  (n = 30) | with intraop FLIP,  adequate (n = 6) | with Intraop FLIP,  inadequate (n = 26) | p-value |
| --- | --- | --- | --- | --- |
| **ES** | 2 (1-3) | 3 (1-4) | 2 (1-3) | 0.49 |
| DI 30 (mm^2^/mmHg) | 3.1 (2.2-4.5) | 2.0 (1.7-2.8) | 2.4 (1.6-4.8) | 0.96 |
| **DI 40** (mm^2^/mmHg) | 4.0 (3.1-6.8) | 3.5 ( 2.6-4.3) | 4.2 (2.6-5.6) | 0.58 |
| DI 50 (mm^2^/mmHg) | 3.8 (3.3-5.9) | 3.3 (3.2-4.0) | 3.4 (2.5-6.1) | 0.96 |
| CSA 30 (mm^2^) | 51.4 (36.4-71.0) | 45.1 (32.8-51.1) | 40.9 (27.1-70.7) | 0.70 |
| CSA 40 (mm^2^) | 102.7 (78.1-156.1) | 82.4 (72.2-108.0) | 90.9 (71.9-126.8) | 0.29 |
| CSA 50 (mm^2^) | 176.8 (134.8-236.3) | 130.4 (110.4-160.8) | 157.5 (94.3-208.0) | 0.53 |
| TBE 1 min (cm) | 3.0 (0-6.2) | 4.8 (2.6-6.2) | 2.0 (0-5.9) | 0.44 |
| TBE 5 min (cm) | 0.5 (0-4.7) | 4.5 (2.1-6.1) | 0 (0-5.2) | 0.26 |
| LES-rp (mmHg) | 10.0 (6.7-15.6)^1^ | 11.0 (8.9-16.7)^2^ | 8.0 (4.9-13.2)^3^ | 0.47 |
| EGD neg/pos | 20/10 | 3/3 | 16/11 | 0.73 |

Median (IQR).^1^ = 27, ^2^ = 5, ^3^ = 22.

a) and b): DI 30, DI 40, DI 50: Distensibiliy at 30, 40 and 50 ml fill volume. CSA 30, CSA 40, CSA 50: Cross-sectional area at 30, 40 and 50 ml fill volume. ES: Eckardt score. TBE: Timed barium esophagogram. LES-rp: Lower esophageal relaxation pressure. EGD: Esophagogastroduodenoscopy.
